# Identification of Putative Serum Autoantibodies Associated with Post-Acute Sequelae of COVID-19 via Comprehensive Protein Array Analysis

**DOI:** 10.3390/ijms26041751

**Published:** 2025-02-19

**Authors:** Yasuyoshi Hatayama, Kei Miyakawa, Yayoi Kimura, Kazuo Horikawa, Kouichi Hirahata, Hirokazu Kimura, Hideaki Kato, Atsushi Goto, Akihide Ryo

**Affiliations:** 1 Department of Virology III, National Institute of Infectious Diseases, Musashimurayama 208-0011, Japan; hatayama@niid.go.jp; 2 Department of Microbiology, Yokohama City University School of Medicine, Yokohama 236-0004, Japan; keim@niid.go.jp; 3 Research Center for Influenza and Respiratory Viruses, National Institute of Infectious Diseases, Musashimurayama 208-0011, Japan; 4 Advanced Medical Research Center, Yokohama City University, Yokohama 236-0004, Japan; ykimura@yokohama-cu.ac.jp (Y.K.); horikawa.kaz.cw@yokohama-cu.ac.jp (K.H.); 5 Hirahata Clinic, Tokyo 150-0002, Japan; koichi.hirahata@gmail.com; 6 Department of Health Science, Gunma Paz University Graduate School of Health Sciences, Takasaki 370-0006, Japan; h-kimura@paz.ac.jp; 7 Infection Prevention and Control Department, Yokohama City University Hospital, Yokohama 236-0004, Japan; ekato@yokohama-cu.ac.jp; 8 Department of Public Health, Yokohama City University School of Medicine, Yokohama 236-0004, Japan; agoto@yokohama-cu.ac.jp

**Keywords:** SARS-CoV-2, autoantibody, protein array, long COVID

## Abstract

Post-acute sequelae of SARS-CoV-2 infection (PASC), commonly known as “Long COVID”, represents a significant clinical challenge characterized by persistent symptoms following acute COVID-19 infection. We conducted a comprehensive retrospective cohort study to identify serum autoantibody biomarkers associated with PASC. Initial screening using a protein bead array comprising approximately 20,000 human proteins identified several candidate PASC-associated autoantibodies. Subsequent validation by enzyme-linked immunosorbent assay (ELISA) in an expanded cohort—consisting of PASC patients, non-PASC COVID-19 convalescents, and pre-pandemic healthy controls—revealed two promising biomarkers: autoantibodies targeting PITX2 and FBXO2. PITX2 autoantibodies demonstrated high accuracy in distinguishing PASC patients from both non-PASC convalescents (area under the curve [AUC] = 0.891) and healthy controls (AUC = 0.866), while FBXO2 autoantibodies showed moderate accuracy (AUC = 0.762 and 0.786, respectively). Notably, the levels of these autoantibodies were associated with several PASC symptoms, including fever, dyspnea, palpitations, loss of appetite, and brain fog. The identification of PITX2 and FBXO2 autoantibodies as biomarkers not only enhances our understanding of PASC pathophysiology but also provides promising candidates for further investigation.

## 1. Introduction

The COVID-19 pandemic has not only highlighted the acute effects of SARS-CoV-2 infection but also its long-term consequences, known as “post-acute sequelae of COVID-19” (PASC) or “Long COVID” [1,2,3]. Recent surveys indicate that approximately 7% of adults and over 1% of children in the United States alone have experienced PASC; it has affected an estimated 15 to 20 million Americans and over 60 million people globally [4]. A certain number of patients have persistent PASC symptoms for months or years after infection, which hinders their reintegration into society and leads to social problems of reduced quality of life and workforce productivity [5].

PASC encompasses a wide range of persistent symptoms, including fatigue, cognitive dysfunction, and cardiopulmonary issues [6]. The heterogeneity of PASC presentations suggests multiple underlying mechanisms, potentially varying between individuals. While the precise pathophysiology of PASC remains elusive, several mechanistic hypotheses have gained prominence, including viral persistence, chronic inflammation, and autoimmune dysregulation [7,8,9,10,11,12].

Several viruses, including Epstein–Barr virus (EBV) and cytomegalovirus (CMV), are known to cause post-viral syndrome. EBV has been shown to be associated with multiple sclerosis through the production of antibodies that cross-react with central nervous system proteins via molecular mimicry of viral proteins [13]. For CMV, it has been reported that infection of vascular endothelial cells leads to exposure of self-antigens from damaged tissues, resulting in the production of autoantibodies [14].

Autoantibodies in plasma and serum in acute COVID-19 have emerged as important mediators in the pathogenesis [15,16]. These autoantibodies target the body’s own tissues through various components of the immune system, coagulation cascade, and tissue-specific antigens [17,18,19,20,21,22]. However, autoantibodies elevated in the acute phase have not been shown to correlate with symptoms in PASC patients. Indeed, despite several exploratory studies investigating PASC-specific autoantibodies, most current studies have not found them in biomarkers that can effectively distinguish patients with full recovery from those with persistent symptoms. This may be due to the persistence of autoantibodies induced by SARS-CoV-2 infection itself during the recovery phase. Furthermore, in the past literature, most studies have targeted a limited range of proteins, such as membrane proteins and immune-related factors, for initial screening [23,24,25,26]. As a result, few reliable serum biomarkers have been identified that could lead to advances in the diagnosis, development of treatments, and prognostic evaluation of PASC. The development and implementation of high-throughput, unbiased proteome-wide screening are needed for the identification of novel and clinically relevant potential biomarker autoantibodies.

To address this critical gap, our study aims to identify novel serum autoantibody biomarkers specifically associated with PASC. We used protein bead array technology combined with high-throughput protein synthesis using wheat cell-free technology [27,28]. The protein bead array enables comprehensive analysis of protein interactions across the human proteome while maintaining the native conformation of proteins. As a primary screening, we performed systematic profiling of autoantibodies in sera from three representative PASC patients with high clinical relevance and extracted candidate autoantibodies by searching for those commonly elevated among patients. We then tested the significance of these autoantibodies as biomarkers in 139 PASC serum samples using ELISA by comparing those from COVID-19 convalescents without lingering symptoms (non-PASC) and pre-pandemic healthy controls (HC). As a result, we identified two distinct biomarkers that could be used for future diagnosis and management of Long COVID patients.

## 2. Results

### 2.1. Development of Autoantibody Screening Methodology

**Figure 1:**
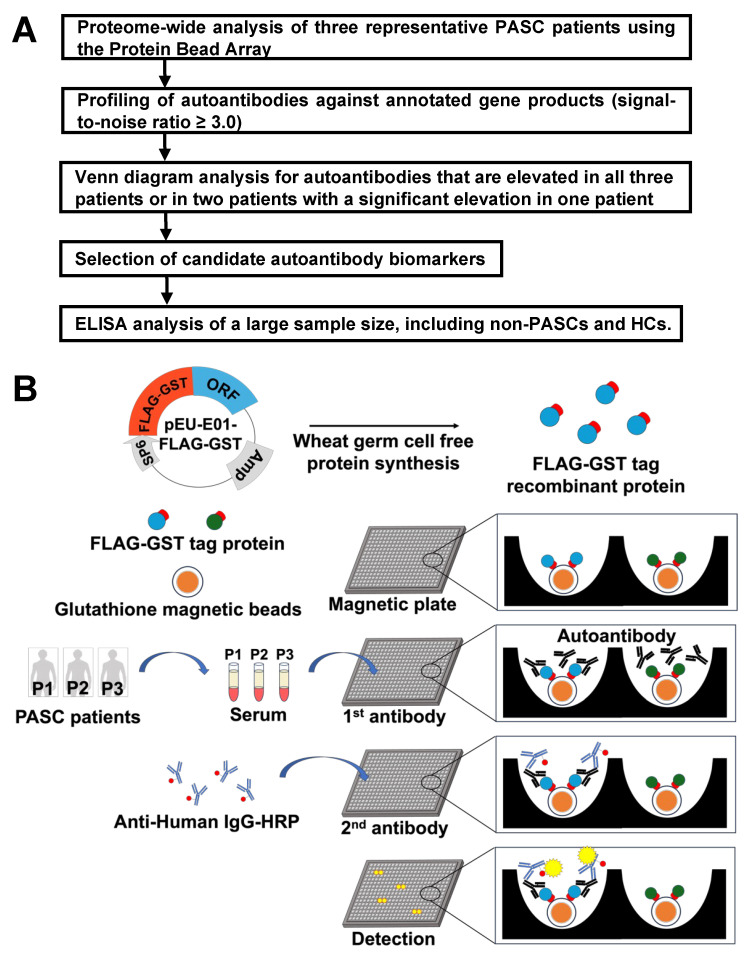
Overview of experimental strategy and protein bead array. (**A**) Flowchart showing the experimental strategy for identifying PASC-specific autoantibodies, from initial screening to validation of candidate biomarkers. (**B**) Schematic of autoantibody screening using the protein bead array platform. Approximately 20,000 full-length human proteins were synthesized in a wheat germ cell-free system. Recombinant proteins were bound to magnetic beads and spotted onto magnetic plates, followed by autoantibody detection with HRP-conjugated anti-human IgG antibodies. Corresponding to the plasmid diagram, red indicates FLAG-GST tags and blue indicates ORFs.

An overview of our workflow is presented in Figure 1A. To establish a robust platform for identifying PASC-specific autoantibodies, a comprehensive screening methodology utilizing a protein bead array was developed. Approximately 20,000 full-length human recombinant proteins were synthesized from FLAG–GST-tagged human full-length cDNA libraries using SP6-driven in vitro transcription and wheat germ cell-free synthesis. The proteins were derived from human cDNA sequences that were characterized by the full-length human cDNA sequencing project (FLJ Project) [29]. Sequence information for both amino acids and nucleotides of open reading frames is available in public human gene and protein databases (HGPDs) [30]. This approach resulted in an array organized in 1536-well plates, with proteins immobilized on magnetic beads in aqueous solution, ensuring maintenance of their native conformational states throughout analysis—a crucial factor for accurate autoantibody detection (Figure 1B).

### 2.2. Identification of Candidate Autoantibodies

**Figure 2:**
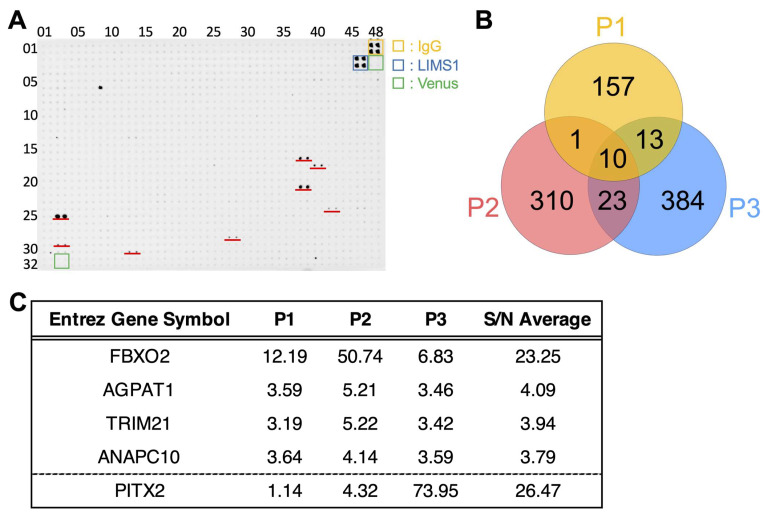
Screening for autoantibodies in PASC serum using protein bead arrays. (**A**) Representative image of the protein bead array. Each protein was spotted in duplicate on 1536-well microplates for autoantibody screening; red lines indicate positive spots. Human IgG and LIMS1 are loaded on the plate as positive controls and Venus as a negative control. (**B**) Venn diagram analysis showing the duplication of autoantibodies in sera from three PASC patients (P1–3), highlighting shared and unique autoantibody signatures. (**C**) Comprehensive list of candidate autoantibodies selected in the initial screening for further validation studies.

Following the establishment of our screening platform, we sought to identify potential PASC-specific autoantibody candidates. Initial proteome-wide profiling of sera from three representative PASC patients with high PASC severity scores revealed a substantial number of autoantibodies. The signal of a representative single-plate array is shown in Figure 2A. LIMS1 has been reported to be detected in healthy human serum [27] and was also carried as a positive control in each plate of the array used in this study (Figure 2A). After unannotated gene products had been excluded, 181, 344, and 430 autoantibodies with S/N ratios ≥ 3.0 were detected in Patients 1, 2, and 3, respectively (Figure 2B, Supplementary Tables S1–S3). Venn diagram analysis revealed ten autoantibodies elevated across all three patients (Figure 2B). From these, four promising candidates (FBXO2, AGPAT1, TRIM21, ANAPC10) that demonstrated particularly robust expression profiles were selected (Figure 2C). Additionally, PITX2 was also included in our candidate pool, as it showed significant elevation in one patient and detectable levels in another (Figure 2C), suggesting potential diagnostic value.

### 2.3. ELISA Validation of Candidate Biomarkers

**Figure 3:**
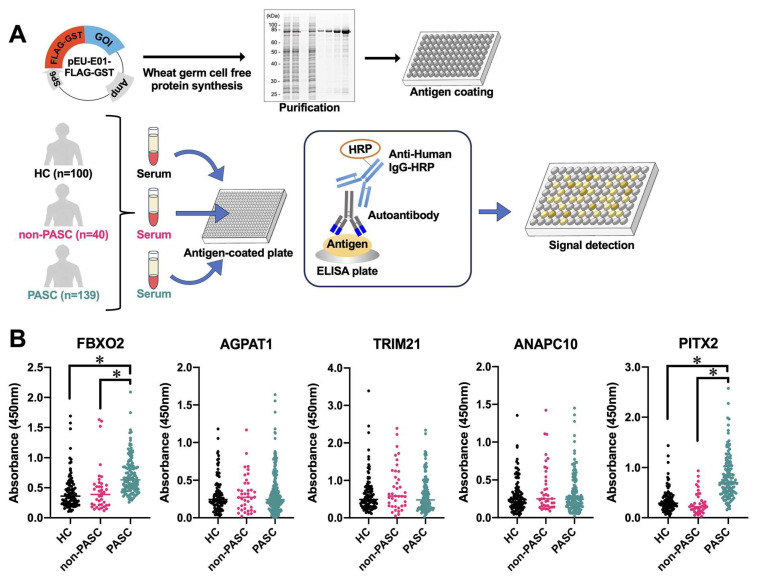
Validation of clinical utility of candidate autoantibodies. (**A**) Schematic diagram of ELISA analysis of autoantibodies induced by PASC. (**B**) Analysis of autoantibody levels (FBXO2, AGPAT1, TRIM21, ANAPC10, and PITX2) in individual samples across three groups: healthy controls (HC), non-PASC COVID-19 patients (non-PASC), and PASC patients. *p*-values were calculated by Mann–Whitney U test. Significance is indicated by *p*-values, as follows: * *p* < 0.01.

To validate our initial findings and assess the clinical utility of these candidate biomarkers, we conducted ELISA analyses using an expanded cohort. Initially, target proteins were synthesized by the wheat cell-free system and immobilized on ELISA plates (Figure 3A). We analyzed serum samples from 139 PASC patients, 40 non-PASC COVID-19 convalescents (non-PASC), and 100 pre-pandemic healthy controls (HC) (Supplementary Tables S4–S6). Comprehensive demographic data (age and sex) were collected for all participants, time since COVID-19 onset was documented for both PASC and non-PASC individuals, and detailed clinical information—including the presence of 14 distinct sequelae symptoms and time elapsed since sequelae onset—was obtained from PASC patients (Supplementary Tables S4–S6). While the sampling period after COVID-19 onset was comparable between PASC and non-PASC groups, significant differences were observed in age distribution between HC and PASC groups, as well as between non-PASC and PASC groups. Additionally, gender distribution differed significantly between HC and PASC groups (Supplementary Table S7). To account for these demographic variations, we performed multiple regression analysis, adjusting for age and sex for each autoantibody measurement (Supplementary Table S8). This analysis revealed that among our candidates, only FBXO2 and PITX2 autoantibodies demonstrated specific elevation in PASC patients, while the other candidates showed no significant differences between groups (Figure 3B).

### 2.4. Biomarker Association Analysis

**Figure 4:**
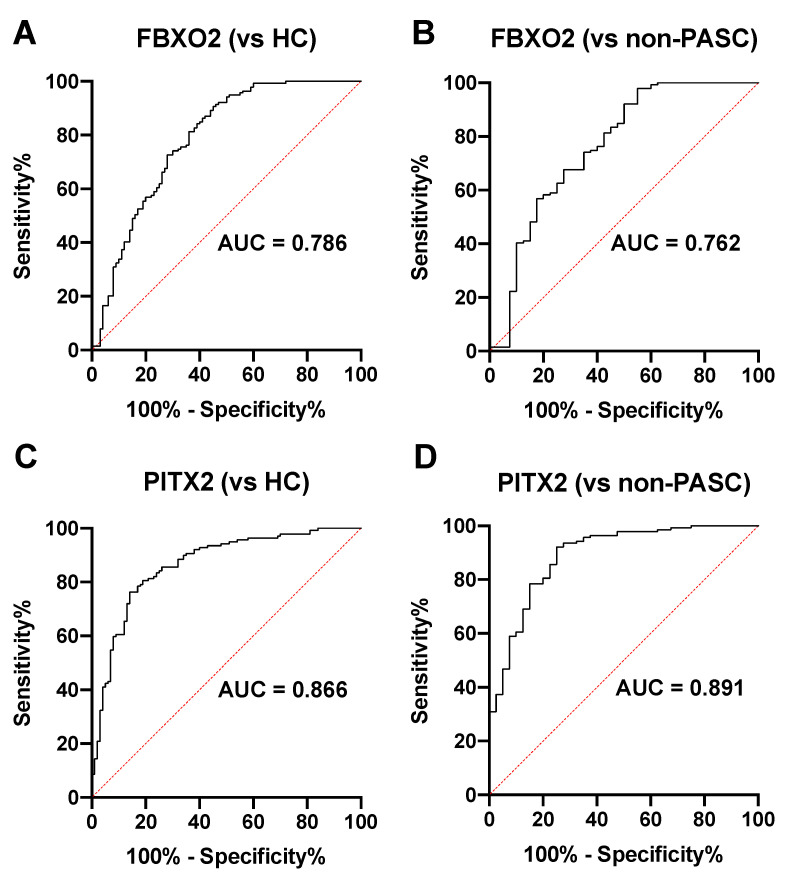
Association between autoantibody levels and PASC status. (**A**,**B**) ROC (receiver operating characteristic) curve analysis of individual FBXO2 autoantibodies in all PASC patients vs. non-PASC patients and healthy controls (HC). The analysis provides AUC (area under the curve) values to assess the potential of these biomarkers. (**C**,**D**) ROC curve analysis of PITX2 autoantibodies in PASC patients vs. those in non-PASC or HC patients, demonstrating the potential utility of these autoantibodies as biomarkers.

To explore relationships between these biomarkers and PASC, we performed ROC curve analyses. FBXO2 antibodies showed an association with PASC status (area under the curve [AUC] = 0.786, 95% CI: 0.725–0.847 vs. HC; AUC = 0.762, 95% CI: 0.666–0.859 vs. non-PASC) (Figure 4A,B, Supplementary Table S9). Similarly, PITX2 antibodies demonstrated potential utility as a biomarker (AUC = 0.866, 95% CI: 0.819–0.914 vs. HC; AUC = 0.891, 95% CI: 0.831–0.951 vs. non-PASC) (Figure 4C,D). For each ROC curve, the values of sensitivity and specificity at the cut-off values determined by the Youden Index and at 90% specificity are shown (Supplementary Table S9). These results together suggest that there may be a potentially meaningful association between these autoantibodies and the incidence of PASC.

### 2.5. Clinical Correlation Analysis

**Figure 5:**
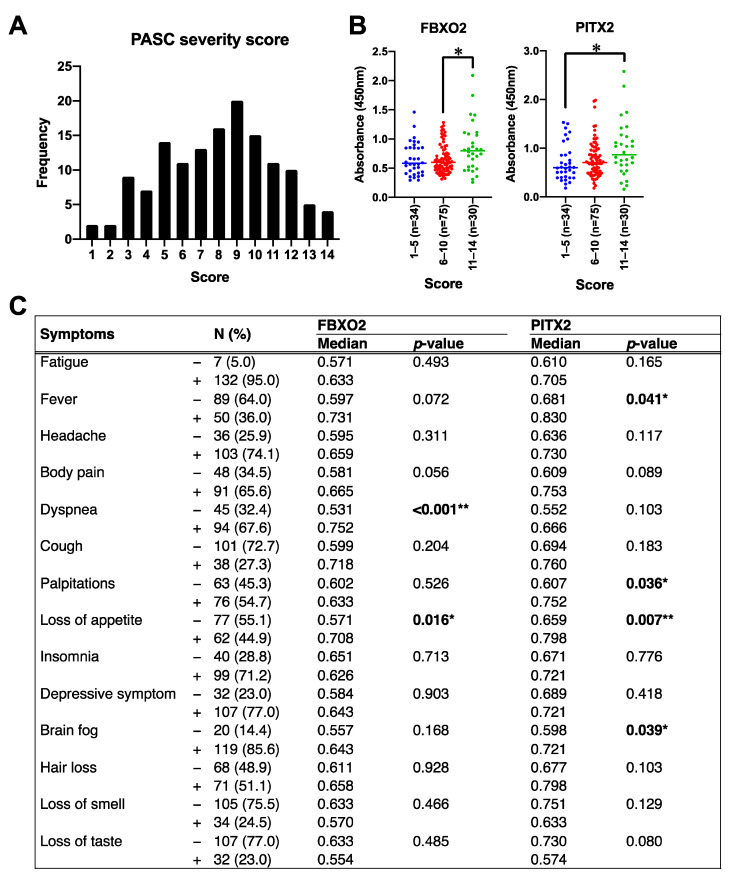
Correlation between autoantibody level and severity in PASC patients. (**A**) Frequency of PASC severity score (maximum 14) among PASC patients. (**B**) Autoantibody levels against PASC severity score; PASC patients were divided into three groups: mild (scores 1–5), moderate (6–10), and severe (11–14), and autoantibody levels in each group were plotted. The *p*-values were calculated by the Mann–Whitney U test. Significance was indicated by the following *p*-values: * *p* < 0.05. (**C**) Comprehensive analysis of relationships between specific PASC-related symptoms and autoantibody levels, presenting the associations between symptom presence/absence and antibody levels. *p*-values were calculated by Mann–Whitney U test. Significance is indicated by the following *p*-values: * *p* < 0.05, ** *p* < 0.01, uncorrected for multiple comparisons. Bold values indicate statistical significance (*p* < 0.05).

To elucidate the clinical relevance of these autoantibodies, their relationship to the severity of PASC and specific symptoms was investigated. PASC severity scores (up to 14 points) for 139 PASC patients are shown in Figure 5A. Patients with higher severity scores showed increased levels of both autoantibodies compared to those with mild or moderate severity (Figure 5B). Analysis of specific symptoms revealed that FBXO2 autoantibody levels were associated with dyspnea and loss of appetite (Figure 5C and Figure S1A), while PITX2 autoantibody levels were associated with fever, palpitations, loss of appetite, and brain fog (Figure 5C and Figure S1B). These finding suggest associations between these autoantibodies and various PASC manifestations, though further investigation is needed to establish their biological and clinical significance.

## 3. Discussion

In this study, we identified previously undetected biomarkers for PASC through comprehensive autoantibody profiling using a protein bead array system. This high-throughput approach, capable of simultaneously screening approximately 20,000 proteins while maintaining their native conformational states, led to the identification of two significant autoantibodies targeting FBXO2 and PITX2. These findings represent a meaningful advancement in our understanding of PASC pathophysiology and may provide potentially promising biomarkers.

The protein bead array system demonstrated several key advantages that enhanced our ability to identify novel biomarkers. By maintaining proteins in solution rather than in a solid state, the system preserves their crucial three-dimensional structures and native conformations [27]. The methodology’s remarkable sensitivity, comparable to conventional immunoblotting techniques, combined with its ability to process small sample volumes, made it particularly effective for autoantibody detection. Furthermore, the system’s comprehensive nature allowed us to obtain detailed autoantibody profiles specific to PASC patients, ultimately distinguishing them from those of both non-PASC and HC patients by combining secondary analysis using ELISA with multiple samples. However, further technical optimization is needed to enhance sensitivity and reduce operational costs. These considerations underscore the importance of strategic implementation of this platform in future studies.

Our analysis revealed elevated levels of autoantibodies against PITX2 and FBXO2 in PASC patients, each with potentially significant implications for disease pathogenesis. PITX2 is a crucial transcription factor involved in cardiac development and function [31,32,33] and may therefore explain certain cardiovascular and neurocognitive manifestations in PASC patients. Notably, we observed a correlation between PITX2 autoantibody levels and palpitation symptoms, suggesting a possible mechanistic link to cardiac pathogenesis. Genome-wide association studies have identified PITX2 as the locus exhibiting the strongest association with atrial fibrillation (AF) [34]. Studies utilizing global heterozygous knockout mice have demonstrated that PITX2 functions as an upstream transcriptional regulator of atrial electrical function, with its deficiency resulting in arrhythmogenic atrial electrical and structural remodeling [35]. Consistent with these findings, atrial cardiomyocytes derived from PITX2-deficient human-induced pluripotent stem cells demonstrate reduced force generation, shortened action potential duration, and hyperpolarized diastolic potential, indicating that PITX2 deficiency is a key contributor to AF susceptibility. Importantly, PITX2 has been shown to regulate oxidative stress responses in cardiac tissue, particularly through modulation of NOX signaling pathways [36]. In SARS-CoV-2 infection, binding of the viral spike protein to ACE2 triggers NOX2 expression through increased angiotensin II levels and enhanced AT1R signaling [37]. The resulting sustained oxidative stress may lead to PITX2 upregulation, potentially contributing to myocardial injury in COVID-19 patients. Our discovery of anti-PITX2 autoantibodies in PASC patients suggests a novel mechanism for cardiovascular pathology, as these autoantibodies could alter both PITX2 function and expression. Further investigation is necessary to elucidate the precise mechanisms through which these autoantibodies affect PITX2 function and to evaluate their potential utility as biomarkers for PASC-associated cardiovascular complications.

Furthermore, PITX2 has been shown to be required for normal development of neurons in the subthalamic nucleus and midbrain in studies by Waite et al. and Martin et al. [38,39]. These studies using mouse models revealed that PITX2 expression is critical during the development of these regions, which are integral components of circuits controlling motor function and cognitive processing. The subthalamic nucleus, in particular, is a key node in the basal ganglia circuit involved in executive function and cognitive control. Recent molecular characterization by Wallén-Mackenzie et al. (2020) has further detailed the complex organization of the subthalamic nucleus, identifying distinct molecular domains and demonstrating its intricate relationship with neighboring glutamatergic and GABAergic structures [40]. The identification of anti-PITX2 autoantibodies in PASC patients suggests a potential mechanism for cognitive dysfunction, particularly “brain fog”. Further research is needed to elucidate the precise mechanisms by which these autoantibodies affect PITX2 function in the mature central nervous system and their potential contribution to PASC-associated cognitive dysfunction.

FBXO2, a member of the F-box protein family, plays a critical role in the ubiquitin–proteasome system (UPS), a fundamental cellular mechanism essential for maintaining protein homeostasis and regulating diverse cellular functions [41]. The protein’s unique characteristic lies in its specific recognition of N-linked glycoproteins, which are predominantly located in the endoplasmic reticulum and crucial for protein folding, quality control, and cellular signaling pathways [42]. Recent research has elucidated the multifaceted roles of FBXO2 across various physiological systems. Preliminary investigations have also suggested a potential correlation between FBXO2 autoantibodies and neurodegenerative conditions, particularly Alzheimer’s disease [43]. Moreover, FBXO2 has been reported to be associated with the regulation of the JAK2/STAT3 pathway and local inflammation [44]. The presence of autoantibodies against FBXO2 may disrupt these regulatory functions, potentially contributing to multi-organ dysfunction and persistent inflammation in PASC. This hypothesis is supported by previous observations of elevated arthritis-related autoantibodies in COVID-19 patients, suggesting an autoimmune component in PASC pathogenesis [45]. Notably, FBXO2 expression is increased in rheumatoid arthritis patients, coinciding with elevated levels of tripartite motif-containing 21 (TRIM21), also known as the SSA/Ro52 antigen [46]. The detection of anti-FBXO2 autoantibodies in PASC patients suggests a breakdown of immune tolerance associated with an ongoing autoimmune process that may play a substantial role in PASC pathophysiology.

The clinical implications of autoantibody production in COVID-19 are significant. Some reports suggest that autoantibodies persist after the acute phase of COVID-19 and contribute to PASC symptoms [47,48,49,50,51,52]. Furthermore, the diversity of autoantibodies observed suggests that COVID-19 may act as a trigger for various autoimmune conditions in susceptible individuals [53]. Several potential mechanisms might explain the development of these autoantibodies in PASC patients. While molecular mimicry between viral and human proteins is one possibility, our preliminary in silico epitope analysis did not reveal sequence similarities between these proteins and SARS-CoV-2. Alternative mechanisms may include the exposure of sequestered antigens through tissue damage and the loss of immune tolerance due to severe lymphocytopenia and immunomodulatory disorders characteristic of COVID-19 [54]. These disturbances in immune regulation could lead to the expansion of autoreactive B-cell clones and persistent autoantibody production [55].

Recent studies have significantly advanced our understanding of autoantibody profiles in PASC patients. Notably, autoantibodies targeting G-protein-coupled receptors, specifically adrenergic receptors (α1 and β2) and muscarinic acetylcholine receptors (M3 and M4), were detected in 60.3% of PASC patients, with 37.2% exhibiting positivity for all investigated receptors [56]. These autoantibodies demonstrated significant correlations with symptom severity, particularly pain intensity and insomnia. Anti-apolipoprotein A-1 IgG (AAA1) has shown a distinct temporal pattern in PASC patients, with a seropositivity rate of 93% at one month post-infection declining to 15% at 12 months. Importantly, AAA1 positivity was predictive of persistent respiratory symptoms [57]. Similarly, anti-nuclear antibodies (ANAs) exhibited a temporal decrease in autoreactivity from 3.99 to 1.55 between 3 and 12 months post-infection. Persistent ANA positivity was associated with several clinical manifestations, including fatigue, dyspnea, and cough severity, as well as sustained inflammation characterized by elevated TNF-α levels [51]. Of particular interest, anti-endothelial cell antibodies (AECA) showed significantly elevated seropositivity rates (52.19%) in patients with olfactory dysfunction, markedly higher than in control groups [58]. These findings collectively highlight the complex autoimmune mechanisms underlying PASC pathophysiology and suggest potential biomarkers for disease monitoring and therapeutic targeting.

The autoantibodies we identified could serve as valuable markers, potentially enabling more accurate disease monitoring and treatment efficacy evaluation. However, several important limitations must be acknowledged. First, serum samples were collected across different medical facilities, potentially introducing bias due to variations in patient demographics and clinical characteristics. Although we observed significant age and gender differences between PASC patients and controls, these known risk factors for PASC [59] were addressed through multiple regression analysis. Although our study incorporated comprehensive patient data, including demographic information (age and gender), temporal parameters (time since COVID-19 onset and sequelae awareness), and symptom profiles at the time of serum collection, several important limitations should be noted. In particular, the lack of data regarding pre-existing medical conditions, which are well-established risk factors for PASC development, represents a significant limitation of our analysis. Collection of this information in future studies will be crucial for better understanding the pathogenesis and pathophysiology of PASC. While our study demonstrates clear associations between specific autoantibodies and PASC, the mechanisms underlying their elevation remain unclear. Several possibilities warrant investigation, including autoantigenicity arising from organ damage, structural homology with viral antigens, and immune dysregulation induced by COVID-19 infection [60,61,62].

The statistical interpretation of our findings requires careful consideration. Although initial analyses revealed significant associations between autoantibodies and clinical symptoms using conventional significance thresholds (*p* < 0.05), these associations did not persist after Bonferroni correction [63] for multiple comparisons (Supplementary Table S10). While the clinical symptoms examined were selected based on established PASC diagnostic guidelines [64,65], suggesting parameter independence, the conservative nature of multiple-comparison corrections may have masked potentially meaningful associations. Despite these limitations, our findings provide compelling evidence for autoimmune mechanisms in PASC pathophysiology and identify promising directions for biomarker development and therapeutic intervention. Future studies, including longitudinal investigations with larger cohorts, will be essential to fully characterize the temporal dynamics of autoantibody production and the role of autoantibodies in disease progression. Additionally, functional studies using in vitro and animal models will be crucial for elucidating the precise mechanisms by which these autoantibodies contribute to PASC pathogenesis.

## 4. Materials and Methods

### 4.1. Samples

Pre-pandemic healthy control (HC) sera were provided by the Biobank Center of Yokohama City University. Sera from non-PASC COVID-19 convalescents (non-PASC) were collected from employees of Yokohama City University who recovered from SARS-CoV-2 infection without sequelae. PASC diagnoses were established according to the World Health Organization (WHO) definition [64]. PASC patient sera were collected from patients who visited the Hirahata Clinic. Information on the presence or absence of subjective symptoms in patients at the time of first visit was obtained from electronic medical records. PASC symptoms included fatigue, fever, headache, body pain, dyspnea, cough, palpitation, loss of appetite, insomnia, depressive symptoms, brain fog, hair loss, loss of taste, and loss of smell [65]. The PASC severity score was defined as the addition of one point for the presence of each of 14 distinct PASC-related symptoms, with a maximum score of 14. Clinical severity was classified as follows: a score of 1–5 as low severity, 6–10 as moderate severity, and 11–14 as high severity.

### 4.2. Analysis of Autoantibodies in Sera of PASC Patients by Protein Bead Array

Protein bead arrays (CellFree Sciences, Yokohama, Japan) were prepared following the manufacturer’s protocol. The arrays were first equilibrated at room temperature for 30 min. After discarding the storage solution, the arrays were washed three times with wash buffer (100 mM NaCl, 0.1% Tween20 in 20 mM Tris-HCl (pH 8.0)) for 10 min each. The reaction buffer was prepared by diluting Synthetic Block (10×) (Invitrogen, Carlsbad, CA, USA) in 1× PBS. Serum samples were diluted to 1:1000 in this buffer, and 10 mL of diluted serum was carefully pipetted into each array well and incubated with gentle shaking at room temperature for 1 h. Following the incubation, the serum diluent was discarded, and the arrays were washed using the previously described washing procedure. Human IgG HRP (5A9) secondary antibody (Abcam, Cambridge, UK) was diluted to 1 µg/mL in the reaction buffer, and 10 mL was dispensed into each array well. The secondary antibody reaction was performed by shaking for 1 h at room temperature. After thorough washing, 10 mL of ImmunoStar LD (FUJIFILM Wako Pure Chemical Corporation, Osaka, Japan) was added to each plate, and spots were subsequently detected using the LAS-3000 imaging system (FUJIFILM Wako Pure Chemical Corporation). The obtained image data were analyzed using ImageJ software (version 1.53k) [66]. Spot quantification was performed for each well on the array, with values normalized by dividing them by the negative control Venus spot present on each array. This normalization method allowed for signal-to-noise ratio (S/N) calculation and inter-plate value equilibration. Given the paired horizontal placement of proteins, a protein was deemed positive when two corresponding dots were detected with S/N ≥ 3. The S/N ratio threshold of ≥3 used in this study was based on a previous study [67,68].

### 4.3. Plasmid Construction

Gene sequences for FBXO2, AGPAT1, TRIM21, ANAPC10A, and PITX2 were obtained from a human complementary DNA (cDNA) library. PCR amplification of each gene was performed using KOD One PCR Master Mix (TOYOBO Co., Ltd., Osaka, Japan) with optimized thermal cycling conditions. The amplified fragments were subsequently cloned into the pEU–E01–FLAG–GST–MCS vector (CellFree Sciences, Yokohama, Japan) using the In-Fusion HD Cloning Kit (Clontech Laboratories, Takara Bio USA, Inc., Palo Alto, CA, USA), targeting the XhoI and KpnI restriction sites.

### 4.4. Cell-Free Protein Synthesis and Purification

In vitro wheat germ cell-free protein synthesis was performed as previously described [69,70]. In vitro transcription was performed using SP6 polymerase. A dialysis cup was filled with a reaction mixture containing WEPRO7240 (CellFree Sciences) wheat germ extract, 250 µL mRNA, and 40 µg/mL creatinine kinase. The mixture was overlaid with 5.5 mL of the SUB-AMIX SGC solution (CellFree Sciences) and then incubated at 15 °C for 72 h using the dialysis overlay method. Here, the upper dialysis cup was placed in a vessel containing 40 mL of SUB-AMIX SGC solution. The synthesized proteins were purified using Glutathione Sepharose 4 Fast Flow (Cytiva, Tokyo, Japan). Thereafter, the bound proteins were eluted with elution buffer (20 mM reduced glutathione in 50 mM Tris-HCl (pH 8.0)). Subsequently, purified proteins were confirmed by Coomassie brilliant blue (CBB) staining using Rapid CBB KANTO 3S (Kanto Chemical, Tokyo, Japan). Purified proteins were stored at −80 °C until use in the assay.

### 4.5. Enzyme-Linked Immunosorbent Assay (ELISA)

Recombinant protein solutions were diluted to 4 µg/mL in 50 mM carbonate buffer (pH 9.6) and added to ELISA plates (Thermo Fisher Scientific, Sunnyvale, CA, USA). To immobilize the proteins, the plates were incubated overnight at 4 °C. Subsequently, the wells were blocked with 2% (*w*/*v*) skim milk in PBS containing 0.05% (*v*/*v*) Tween-20 (PBS-T) for 1 h at 37 °C and washed three times with PBS-T. Next, 50 μL of diluted serum (1:100 in 0.2% skim milk) was added to each well, and the plates were incubated for 1 h at 25 °C. After washing three times with PBS-T, 100 μL of anti-human IgG conjugated to HRP were added to each well, and the plates were incubated at 25 °C for 1 h. After washing three times with PBS-T, 100 μL of the TMB substrate solution (SeraCare Life Sciences, Inc., Milford, MA, USA) was added, and the color developed for 15 min. Then, stopping solution was added, and absorbance was measured at 450 nm using the GloMax Discover System (Promega Corporation, Madison, WI, USA). Linearity of measurements was established using serial dilutions of FLAG antibody (from 10^−3^ to 3.9 × 10^−6^ with two-fold dilution steps) to generate standard curves and perform inter-plate normalization. All assays were performed in duplicate for each sample.

### 4.6. Statistical Analysis

Statistical analyses were conducted using GraphPad Prism software (version 8.4.3; GraphPad Software Inc., San Diego, CA, USA). Comparisons between continuous variables of two groups were performed using a two-tailed Mann–Whitney U test. Statistical significance was defined as a *p*-value less than 0.05 (*p* < 0.05). To account for multiple comparisons in the analysis of associations between autoantibodies and clinical manifestations, Bonferroni correction was applied by adjusting the significance threshold to *p* < (adjusted value = 0.05/number of comparisons) [63].

Multiple regression analysis was performed using EZR (Saitama Medical Center, Jichi Medical University, Saitama, Japan) [71], which is a graphical user interface for R (The R Foundation for Statistical Computing, Vienna, Austria, version 4.4.2), to adjust for the effects of age and gender on autoantibody levels. The regression models incorporated group status (PASC and non-PASC, with HC as reference group), age (continuous variable), and gender (male as reference) as independent variables, as previously described [72,73]. The antibody levels were then adjusted for age and gender using coefficients obtained from the regression analysis. The adjustment was performed using the following formula: for males, the adjusted value was calculated as Y − a(Age_i − Age_mean), and for females, as Y − a(Age_i − Age_mean) − b, where Y is the measured antibody level, a is the age coefficient, Age_i is the individual’s age, Age_mean is the mean age of the study population, and b is the gender coefficient representing the female effect.

## Data Availability

The original contributions presented in this study are included in the article/Supplementary Material. Further inquiries can be directed to the corresponding author.

## Supplementary Materials

The supporting information can be downloaded at: https://www.mdpi.com/article/10.3390/ijms26041751/s1.

## References

[1] Nabavi N. (2020). Long COVID: How to Define It and How to Manage It. BMJ.

[2] Nalbandian A., Sehgal K., Gupta A., Madhavan M.V., McGroder C., Stevens J.S., Cook J.R., Nordvig A.S., Shalev D., Sehrawat T.S. (2021). Post-Acute COVID-19 Syndrome. Nat. Med..

[3] Subramanian A., Nirantharakumar K., Hughes S., Myles P., Williams T., Gokhale K.M., Taverner T., Chandan J.S., Brown K., Simms-Williams N. (2022). Symptoms and Risk Factors for Long COVID in Non-Hospitalized Adults. Nat. Med..

[4] Ely E.W., Brown L.M., Fineberg H.V. (2024). Long COVID Defined. N. Engl. J. Med..

[5] Cutler D.M. (2022). The Costs of Long COVID. JAMA Health Forum.

[6] Carfì A., Bernabei R., Landi F. (2020). Persistent Symptoms in Patients After Acute COVID-19. JAMA.

[7] Karlsson A.C., Humbert M., Buggert M. (2020). The Known Unknowns of T Cell Immunity to COVID-19. Sci. Immunol..

[8] Li Q., Zheng X.S., Shen X.R., Si H.R., Wang X., Wang Q., Li B., Zhang W., Zhu Y., Jiang R.-D. (2020). Prolonged Shedding of Severe Acute Respiratory Syndrome Coronavirus 2 in Patients with COVID-19. Emerg. Microbes Infect..

[9] Carmo A., Pereira-Vaz J., Mota V., Mendes A., Morais C., da Silva A.C., Camilo E., Pinto C.S., Cunha E., Pereira J. (2020). Clearance and Persistence of SARS-CoV-2 RNA in Patients with COVID-19. J. Med. Virol..

[10] Brodin P., Casari G., Townsend L., O’Farrelly C., Tancevski I., Löffler-Ragg J., Mogensen T.H., Casanova J.L., Abel L., Aiuti A. (2022). Studying Severe Long COVID to Understand Post-Infectious Disorders beyond COVID-19. Nat. Med..

[11] Wu Y., Guo C., Tang L., Hong Z., Zhou J., Dong X., Yin H., Xiao Q., Tang Y., Qu X. (2020). Prolonged Presence of SARS-CoV-2 Viral RNA in Faecal Samples. Lancet Gastroenterol. Hepatol..

[12] Glynne P., Tahmasebi N., Gant V., Gupta R. (2022). Long COVID Following Mild SARS-CoV-2 Infection: Characteristic T Cell Alterations and Response to Antihistamines. J. Investig. Med..

[13] Lanz T.V., Brewer R.C., Ho P.P., Moon J.S., Jude K.M., Fernandez D., Fernandes R.A., Gomez A.M., Nadj G.S., Bartley C.M. (2022). Clonally Expanded B Cells in Multiple Sclerosis Bind EBV EBNA1 and GlialCAM. Nature.

[14] Hebart H., Einsele H., Klein R., Fischer I., Bühler S., Dietz K., Jahn G., Berg P.A., Kanz L., Müller C.A. (1996). CMV Infection after Allogeneic Bone Marrow Transplantation Is Associated with the Occurrence of Various Autoantibodies and Monoclonal Gammopathies. Br. J. Haematol..

[15] Dotan A., Muller S., Kanduc D., David P., Halpert G., Shoenfeld Y. (2021). The SARS-CoV-2 as an Instrumental Trigger of Autoimmunity. Autoimmun. Rev..

[16] Chang S.E., Feng A., Meng W., Apostolidis S.A., Mack E., Artandi M., Barman L., Bennett K., Chakraborty S., Chang I. (2021). New-Onset IgG Autoantibodies in Hospitalized Patients with COVID-19. Nat. Commun..

[17] Pretorius E., Vlok M., Venter C., Bezuidenhout J.A., Laubscher G.J., Steenkamp J., Kell D.B. (2021). Persistent Clotting Protein Pathology in Long COVID/Post-Acute Sequelae of COVID-19 (PASC) Is Accompanied by Increased Levels of Antiplasmin. Cardiovasc. Diabetol..

[18] Kreye J., Reincke S.M., Prüss H. (2020). Do Cross-Reactive Antibodies Cause Neuropathology in COVID-19?. Nat. Rev. Immunol..

[19] Galeotti C., Bayry J. (2020). Autoimmune and Inflammatory Diseases Following COVID-19. Nat. Rev. Rheumatol..

[20] Bastard P., Gervais A., Voyer T.L., Rosain J., Philippot Q., Manry J., Michailidis E., Hoffmann H.H., Eto S., Garcia-Prat M. (2021). Autoantibodies Neutralizing Type I IFNs Are Present in ~4% of Uninfected Individuals over 70 Years Old and Account for ~20% of COVID-19 Deaths. Sci. Immunol..

[21] Zuniga M., Gomes C., Carsons S.E., Bender M.T., Cotzia P., Miao Q.R., Lee D.C., Rodriguez A. (2021). Autoimmunity to Annexin A2 Predicts Mortality among Hospitalised COVID-19 Patients. Eur. Respir. J..

[22] Zuo Y., Estes S.K., Ali R.A., Gandhi A.A., Yalavarthi S., Shi H., Sule G., Gockman K., Madison J.A., Zuo M. (2020). Prothrombotic Autoantibodies in Serum from Patients Hospitalized with COVID-19. Sci. Transl. Med..

[23] Klein J., Wood J., Jaycox J.R., Dhodapkar R.M., Lu P., Gehlhausen J.R., Tabachnikova A., Greene K., Tabacof L., Malik A.A. (2023). Distinguishing Features of Long COVID Identified through Immune Profiling. Nature.

[24] Wang E.Y., Mao T., Klein J., Dai Y., Huck J.D., Jaycox J.R., Liu F., Zhou T., Israelow B., Wong P. (2021). Diverse Functional Autoantibodies in Patients with COVID-19. Nature.

[25] Zhao J., Schank M., Wang L., Dang X., Cao D., Khanal S., Nguyen L.N.T., Zhang Y., Wu X.Y., Adkins J.L. (2022). Plasma Biomarkers for Systemic Inflammation in COVID-19 Survivors. Proteom. Clin. Appl..

[26] Peluso M.J., Lu S., Tang A.F., Durstenfeld M.S., Ho H.E., Goldberg S.A., Forman C.A., Munter S.E., Hoh R., Tai V. (2021). Markers of Immune Activation and Inflammation in Individuals with Postacute Sequelae of Severe Acute Respiratory Syndrome Coronavirus 2 Infection. J. Infect. Dis..

[27] Yoshitaka K., Goshima N. (2014). Development of a Protein Array for Autoantibody Profiling of Blood. Synthesiology.

[28] Morishita R., Sugiyama S., Denda M., Tokunaga S., Kido K., Shioya R., Ozawa S., Sawasaki T. (2019). CF-PA2Vtech: A Cell-Free Human Protein Array Technology for Antibody Validation against Human Proteins. Sci. Rep..

[29] Maruyama Y., Wakamatsu A., Kawamura Y., Kimura K., Yamamoto J.I., Nishikawa T., Kisu Y., Sugano S., Goshima N., Isogai T. (2008). Human Gene and Protein Database (HGPD): A Novel Database Presenting a Large Quantity of Experiment-Based Results in Human Proteomics. Nucleic Acids Res..

[30] Human Gene and Protein Database (HGPD). https://hgpd.lifesciencedb.jp/cgi/index.cgi.

[31] Hill M.C., Kadow Z.A., Li L., Tran T.T., Wythe J.D., Martin J.F. (2019). A Cellular Atlas of Pitx2-Dependent Cardiac Development. Development.

[32] Hernandez-Torres F., Rodríguez-Outeiriño L., Franco D., Aranega A.E. (2017). Pitx2 in Embryonic and Adult Myogenesis. Front. Cell Dev. Biol..

[33] Tran T.Q., Kioussi C. (2021). Pitx Genes in Development and Disease. Cell. Mol. Life Sci..

[34] Zheng S.L., Henry A., Cannie D., Lee M., Miller D., McGurk K.A., Bond I., Xu X., Issa H., Francis C. (2024). Genome-Wide Association Analysis Provides Insights into the Molecular Etiology of Dilated Cardiomyopathy. Nat. Genet..

[35] Kirchhof P., Kahr P.C., Kaese S., Piccini I., Vokshi I., Scheld H.H., Rotering H., Fortmueller L., Laakmann S., Verheule S. (2011). PITX2c Is Expressed in the Adult Left Atrium, and Reducing Pitx2c Expression Promotes Atrial Fibrillation Inducibility and Complex Changes in Gene Expression. Circ. Cardiovasc. Genet..

[36] Sridhar A., DeSantiago J., Chen H., Pavel M.A., Ly O., Owais A., Barney M., Jousma J., Nukala S.B., Abdelhady K. (2024). Modulation of NOX2 Causes Obesity-Mediated Atrial Fibrillation. J. Clin. Investig..

[37] Sindona C., Schepici G., Contestabile V., Bramanti P., Mazzon E. (2021). NOX2 Activation in COVID-19: Possible Implications for Neurodegenerative Diseases. Medicina.

[38] Waite M.R., Martin D.M. (2015). Axial Level-Specific Regulation of Neuronal Development: Lessons from PITX2. J. Neurosci. Res..

[39] Martin D.M., Skidmore J.M., Philips S.T., Vieira C., Gage P.J., Condie B.G., Raphael Y., Martinez S., Camper S.A. (2004). PITX2 Is Required for Normal Development of Neurons in the Mouse Subthalamic Nucleus and Midbrain. Dev. Biol..

[40] Wallén-Mackenzie Å., Dumas S., Papathanou M., Martis Thiele M.M., Vlcek B., König N., Björklund Å.K. (2020). Spatio-Molecular Domains Identified in the Mouse Subthalamic Nucleus and Neighboring Glutamatergic and GABAergic Brain Structures. Commun. Biol..

[41] Tekcham D.S., Chen D., Liu Y., Ling T., Zhang Y., Chen H., Wang W., Otkur W., Qi H., Xia T. (2020). F-Box Proteins and Cancer: An Update from Functional and Regulatory Mechanism to Therapeutic Clinical Prospects. Theranostics.

[42] Nelson R.F., Glenn K.A., Zhang Y., Wen H., Knutson T., Gouvion C.M., Robinson B.K., Zhou Z., Yang B., Smith R.J.H. (2007). Selective Cochlear Degeneration in Mice Lacking the F-Box Protein, Fbx2, a Glycoprotein-Specific Ubiquitin Ligase Subunit. J. Neurosci..

[43] Atkin G., Hunt J., Minakawa E., Sharkey L., Tipper N., Tennant W., Paulson H.L. (2014). F-Box Only Protein 2 (Fbxo2) Regulates Amyloid Precursor Protein Levels and Processing. J. Biol. Chem..

[44] Zhao X., Guo W., Zou L., Hu B. (2020). FBXO2 Modulates STAT3 Signaling to Regulate Proliferation and Tumorigenicity of Osteosarcoma Cells. Cancer Cell Int..

[45] Vlachoyiannopoulos P.G., Magira E., Alexopoulos H., Jahaj E., Theophilopoulou K., Kotanidou A., Tzioufas A.G. (2020). Autoantibodies Related to Systemic Autoimmune Rheumatic Diseases in Severely Ill Patients with COVID-19. Ann. Rheum. Dis..

[46] Mizutani Y., Matsuoka K., Takeda H., Shiogama K., Inada K.I., Hayakawa K., Yamada H., Miyazaki T., Sawasaki T., Endo Y. (2013). Novel Approach to Identifying Autoantibodies in Rheumatoid Synovitis with a Biotinylated Human Autoantigen Library and the Enzyme-Labeled Antigen Method. J. Immunol. Methods.

[47] Proal A.D., VanElzakker M.B. (2021). Long COVID or Post-Acute Sequelae of COVID-19 (PASC): An Overview of Biological Factors That May Contribute to Persistent Symptoms. Front. Microbiol..

[48] Sotzny F., Filgueiras I.S., Kedor C., Freitag H., Wittke K., Bauer S., Sepúlveda N., Mathias da Fonseca D.L., Baiocchi G.C., Marques A.H.C. (2022). Dysregulated Autoantibodies Targeting Vaso- and Immunoregulatory Receptors in Post COVID Syndrome Correlate with Symptom Severity. Front. Immunol..

[49] Acosta-Ampudia Y., Monsalve D.M., Rojas M., Rodriguez Y., Zapata E., Ramirez-Santana C., Anaya J.M. (2022). Persistent Autoimmune Activation and Proinflammatory State in Post-Coronavirus Disease 2019 Syndrome. J. Infect. Dis..

[50] Seibert F.S., Stervbo U., Wiemers L., Skrzypczyk S., Hogeweg M., Bertram S., Kurek J., Anft M., Westhoff T.H., Babel N. (2023). Severity of Neurological Long-COVID Symptoms Correlates with Increased Level of Autoantibodies Targeting Vasoregulatory and Autonomic Nervous System Receptors. Autoimmun. Rev..

[51] Son K., Jamil R., Chowdhury A., Mukherjee M., Venegas C., Miyasaki K., Zhang K., Patel Z., Salter B., Yan Yuen A.C. (2023). Circulating Anti-Nuclear Autoantibodies in COVID-19 Survivors Predict Long COVID Symptoms. Eur. Respir. J..

[52] Jernbom A.F., Skoglund L., Pin E., Sjöberg R., Tegel H., Hober S., Rostami E., Rasmusson A., Cunningham J.L., Havervall S. (2024). Prevalent and Persistent New-Onset Autoantibodies in Mild to Severe COVID-19. Nat. Commun..

[53] Ehrenfeld M., Tincani A., Andreoli L., Cattalini M., Greenbaum A., Kanduc D., Alijotas-Reig J., Zinserling V., Semenova N., Amital H. (2020). Covid-19 and Autoimmunity. Autoimmun. Rev..

[54] Lyons-Weiler J. (2020). Pathogenic Priming Likely Contributes to Serious and Critical Illness and Mortality in COVID-19 via Autoimmunity. J. Transl. Autoimmun..

[55] Woodruff M.C., Ramonell R.P., Nguyen D.C., Cashman K.S., Saini A.S., Haddad N.S., Ley A.M., Kyu S., Howell J.C., Ozturk T. (2020). Extrafollicular B Cell Responses Correlate with Neutralizing Antibodies and Morbidity in COVID-19. Nat. Immunol..

[56] Ceccarini M.R., Bonetti G., Medori M.C., Dhuli K., Tezzele S., Micheletti C., Maltese P.E., Cecchin S., Donato K., Fioretti F. (2023). Autoantibodies in Patients with Post-COVID Syndrome: A Possible Link with Severity?. Eur. Rev. Med. Pharmacol. Sci..

[57] L’Huillier A.G., Pagano S., Baggio S., Meyer B., Andrey D.O., Nehme M., Guessous I., Eberhardt C.S., Huttner A., Posfay-Barbe K.M. (2022). Autoantibodies against Apolipoprotein A-1 after COVID-19 Predict Symptoms Persistence. Eur. J. Clin. Invest..

[58] Fiorelli D., Francavilla B., Velletrani G., Maurantonio S., Passali F.M., Bernardini S., Di Girolamo S., Nuccetelli M. (2024). Autoantibody Profiles Assessment in Individuals with Persistent Olfactory Impairment Following SARS-CoV-2 Infection. Int. Immunopharmacol..

[59] Notarte K.I., de Oliveira M.H.S., Peligro P.J., Velasco J.V., Macaranas I., Ver A.T., Pangilinan F.C., Pastrana A., Goldrich N., Kavteladze D. (2022). Age, Sex and Previous Comorbidities as Risk Factors Not Associated with SARS-CoV-2 Infection for Long COVID-19: A Systematic Review and Meta-Analysis. J. Clin. Med..

[60] Mohkhedkar M., Venigalla S.S.K., Janakiraman V. (2021). Untangling COVID-19 and Autoimmunity: Identification of Plausible Targets Suggests Multi Organ Involvement. Mol. Immunol..

[61] Lee J.S., Park S., Jeong H.W., Ahn J.Y., Choi S.J., Lee H., Choi B., Nam S.K., Sa M., Kwon J.S. (2020). Immunophenotyping of COVID-19 and Influenza Highlights the Role of Type I Interferons in Development of Severe COVID-19. Sci. Immunol..

[62] Lucchese G., Flöel A. (2020). Molecular Mimicry between SARS-CoV-2 and Respiratory Pacemaker Neurons. Autoimmun. Rev..

[63] Curtin F., Schulz P. (1998). Multiple Correlations and Bonferroni’s Correction. Biol. Psychiatry.

[64] (2021). A Clinical Case Definition of Post COVID-19 Condition by a Delphi Consensus.

[65] Hirahata K., Nawa N., Fujiwara T. (2022). Characteristics of Long COVID: Cases from the First to the Fifth Wave in Greater Tokyo, Japan. J. Clin. Med..

[66] Schneider C.A., Rasband W.S., Eliceiri K.W. (2012). NIH Image to ImageJ: 25 Years of Image Analysis. Nat. Methods.

[67] Zhang J.H., Chung T.D.Y., Oldenburg K.R. (1999). A Simple Statistical Parameter for Use in Evaluation and Validation of High Throughput Screening Assays. J. Biomol. Screen..

[68] van der Meulen P.M., Barendregt A.M., Cuadrado E., Magro-Checa C., Steup-Beekman G.M., Meinema D.S., Merlijn Van den Berg J., Li Q.Z., Baars P.A., Wouters D. (2017). Protein Array Autoantibody Profiles to Determine Diagnostic Markers for Neuropsychiatric Systemic Lupus Erythematosus. Rheumatology.

[69] Matsunaga S., Masaoka T., Sawasaki T., Morishita R., Iwatani Y., Tatsumi M., Endo Y., Yamamoto N., Sugiura W., Ryo A. (2015). A Cell-Free Enzymatic Activity Assay for the Evaluation of HIV-1 Drug Resistance to Protease Inhibitors. Front. Microbiol..

[70] Yamaoka Y., Matsunaga S., Jeremiah S.S., Nishi M., Miyakawa K., Morita T., Khatun H., Shimizu H., Okabe N., Kimura H. (2021). Zika Virus Protease Induces Caspase-Independent Pyroptotic Cell Death by Directly Cleaving Gasdermin D. Biochem. Biophys. Res. Commun..

[71] Kanda Y. (2013). Investigation of the Freely Available Easy-to-Use Software “EZR” for Medical Statistics. Bone Marrow Transplant.

[72] Schneider A., Hommel G., Blettner M. (2010). Linear Regression Analysis: Part 14 of a Series on Evaluation of Scientific Publications. Dtsch. Arztebl. Int..

[73] Fonseca D.L.M., Filgueiras I.S., Marques A.H.C., Vojdani E., Halpert G., Ostrinski Y., Baiocchi G.C., Plaça D.R., Freire P.P., Pour S.Z. (2023). Severe COVID-19 Patients Exhibit Elevated Levels of Autoantibodies Targeting Cardiolipin and Platelet Glycoprotein with Age: A Systems Biology Approach. npj Aging.

