# Supplementary material for: Identification of Putative Serum Autoantibodies Associated with Post-Acute Sequelae of COVID-19 via Comprehensive Protein Array Analysis
Source: Int J Mol Sci. 2025 Feb 19;26(4):1751. doi: 10.3390/ijms26041751 (PMC11855120; doi:10.3390/ijms26041751)
Supplement: Supplementary file 1 [file ijms-26-01751-s001.zip › Sup Figure proof.pdf]

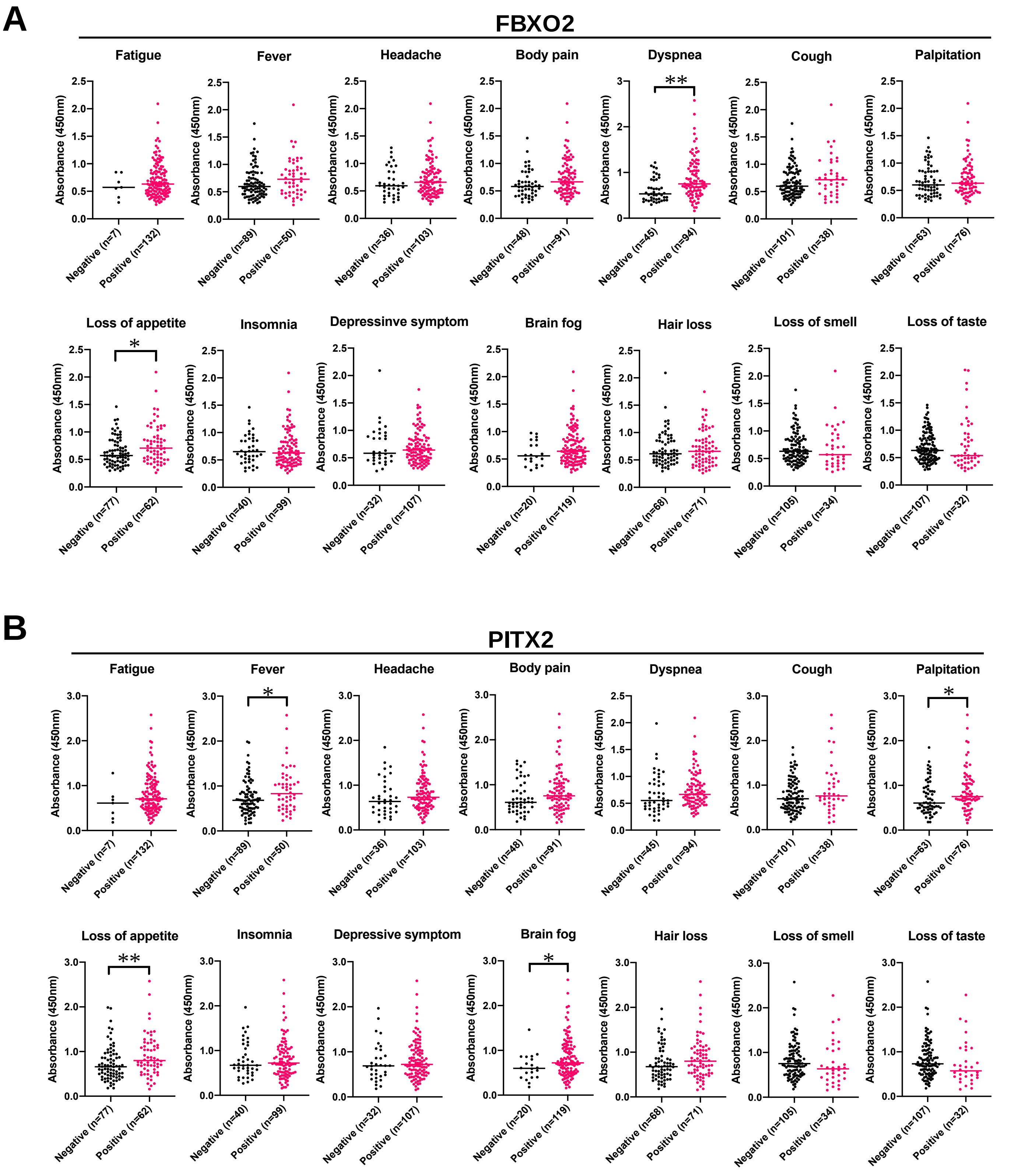

Figure.S1 Analysis of autoantibody levels in relation to PASC clinical manifestations. Association between FBOX2 (A) and PITX2 (B) autoantibody levels and the presence of various PACS-related symptoms. p-values were calculated by Mann-Whitney U test. Significance is indicated by the following p-values: *p<0.05, **p<0.01, uncorrected for multiple comparisons.
